# Care Managers Have Few Options for Home Modification Because They Are Not Specified in Architecture

**DOI:** 10.1093/geroni/igaa057.641

**Published:** 2020-12-16

**Authors:** Kazunori Yoshida, Yoritaka Harazono, Toko Funaki, Akiko Nishino

**Affiliations:** The University of Tokyo, Tokyo, Japan

## Abstract

In this paper, we aim to clarify the cause of the difficulty in home modification. The aging society becomes larger where older people have difficulty in living home because of weakened body functions. To maintain quality of life, it is important to modify houses. In Japan, home modification is conducted by care managers, who are originally from nurses, helpers, and so on. However, to modify houses, we hypothesized that it is needed to have knowledge about not only body function but also architecture. Because of this, home modification should be difficult for care managers. For this problem, we aim to clarify the difficulty in home modification. In November 2018, we took part in the teaching course for care managers about home modification and asked care managers the number of home modification they conducted and what they have difficulty in. As a result, we asked for 57 care managers, who have experience as care managers for 39 months in average. Home modification was mainly conducted for setting handrails (four for a care manager in average). It was also revealed that experience of modification for handrails and doors are larger when the experience of care manager becomes longer, but other modification is not the case. The care managers told us that they cannot understand architecture. This result indicates that care managers cannot think of many options for modification because of their little knowledge about architecture. Therefore, it should be needed to combine the architects and care managers for appropriate home modification.

